# Immune disruptions and night shift work in hospital healthcare professionals: The intricate effects of social jet-lag and sleep debt

**DOI:** 10.3389/fimmu.2022.939829

**Published:** 2022-09-09

**Authors:** Brice Faraut, Emilie Cordina-Duverger, Guillen Aristizabal, Catherine Drogou, Caroline Gauriau, Fabien Sauvet, Francis Lévi, Damien Léger, Pascal Guénel

**Affiliations:** ^1^ Université Paris Cité, VIFASOM (UPR 7330 Vigilance Fatigue, Sommeil et Santé Publique), Paris, France; ^2^ APHP, APHP-Centre Université de Paris, Hôtel Dieu, Centre du Sommeil et de La Vigilance, Paris, France; ^3^ Inserm, CESP (Center for research in Epidemiology and Population Health), Team Exposome and Heredity, University Paris-Saclay, Gustave-Roussy, Villejuif, France; ^4^ Institut de Recherche Biomédicale des Armées (IRBA), Unité Fatigue et Vigilance, Brétigny sur Orge, France; ^5^ UPR “Chronothérapie, Cancers, et Transplantation”, Faculté de Médecine, Université Paris-Saclay, Villejuif, France; ^6^ Hepato-Biliary Center, Hôpital Paul Brousse, Villejuif, France; ^7^ Cancer Chronotherapy Team, Cancer Research Centre, Division of Biomedical Sciences, Warwick Medical School, Coventry, United Kingdom

**Keywords:** night shift work, sleep debt, social-jet lag, circulating leucocytes, interleukin-6, circadian disruption, hospital workers

## Abstract

**Objectives:**

We aimed to examine the effects of circadian and sleep rhythm disruptions on immune biomarkers among hospital healthcare professionals working night shifts and rotating day shifts.

**Methods:**

Hospital nurses working either as permanent night shifters (n=95) or as day shifters rotating between morning and afternoon shifts (n=96) kept a daily diary on their sleep and work schedules over a full working week. Blood samples were collected at the beginning and end of the last shift during the week, and participants were categorized into three groups based on work shift: morning shift (39 day shifters sampled at 7:00 and 14:00), afternoon shift (57 day shifters sampled at 14:00 and 21:00), and night shift (95 night shifters sampled at 21:00 and 7:00). Circulating blood counts in immune cells, interleukin-6 and C-reactive protein concentrations as well as total sleep time per 24 hours during work days (TST24w) and free days (TST24f), sleep debt (TST24f — TST24w) and social jet-lag (a behavioral proxy of circadian misalignment) were assessed.

**Results:**

Compared with day shifters, night shifters had shorter sleep duration (TST24w=5.4 ± 1.4h), greater sleep debt (3.2 ± 1.4 h) and social jet-lag (6.7 ± 2.4 h). Variations of immune biomarkers concentrations were consistent with the expected diurnal variations among day shifters (i.e., low level in the morning, increase during the day, peak value in the evening). By contrast, in night shifters, blood concentrations of total lymphocytes, T-helper cells, cytotoxic T-cells, memory B-cells and interleukin-6 were lower at 21:00, increased during the night, and reached higher values at 7:00. Multivariate analyses ruled out significant impact of TST24w, sleep debt, and social jet-lag on immune biomarkers concentrations among day shifters. In contrast, among night shifters, multivariate analyses indicated a combined effect of total sleep time (TST24w), sleep debt and social jet-lag for total lymphocytes and T-helper cells but only a social jet-lag effect for interleukin-6 and a single total sleep time effect for neutrophil and B-Cells.

**Conclusions:**

Altogether, our results point to intricate response patterns of immune rhythms to circadian misalignment and sleep debt in night shifters. Specifically, these altered pattern expressions of immune cells may increase vulnerability to infections and reduce vaccination efficiency in night workers.

## Introduction

Shift work including night work involves 15-20% of the total working population in the world (WHO, 2020) (1). Changes in work timing schedules frequently disrupt the tight links between the circadian rhythms that drive and regulate sleep, and sleep processes. Thus, many shift workers sleep at the “wrong” physiological time, because of the odd timing of imposed sleep episodes related to the work schedules. Indeed, shift and night workers are chronically poor sleepers, with shortened sleep durations and have to cope both with sleep debt and with circadian desynchronization (2–4). The role of the desynchronization of the circadian clock from night shift work begins to be well established for the pathogenesis of breast and other cancers, as well as for metabolic, cardiovascular and infectious diseases (5–10). In addition, in order to compensate for the extended “daily” wakefulness durations, shift workers frequently ‘catch up’ on sleep on free days. Increasing laboratory-based scientific evidence indicates that the repetition of a pattern of sleep restriction and recovery *per se* disrupts several physiological homeostatic and circadian mechanisms including insulin resistance, pain-related symptoms or stress and immune dysregulations (e.g. cortisol and pro-inflammatory cytokines) (11–13).

The level of immune cells during a 24-h day depends both on circadian and homeostatic sleep regulations through hormone and neural innervations (14). Under regular sleep-wake conditions, human leucocyte subsets show circadian rhythms with peak counts late afternoon (Natural-Killer (NK)- Cells and neutrophils) or late evening (lymphocytes, monocytes) and decreasing circulating counts while sleeping during the night (14, 15). These changes indicate redistribution between the vascular compartment and extravascular lymphoid tissues supporting enhanced process of immune memory consolidation during sleep when acute exposure to environmental immune challenge is usually less frequent (16, 17). Compelling evidence from simulated night shift work links circadian misalignment and sleep restriction to suppressed, blunted and/or shifted immune-inflammatory rhythms (18–21). Hence, the immune and inflammatory systems could act as physiological mediators for the damaging health effects imposed by shift work exposure (22). What’s more, the efficiency of immune responses to pathogens does change with time of the day (23, 24). For instance, Bacillus Calmette–Guérin (BCG) vaccination induced a more robust trained immunity and adaptive responses following morning as compared to evening vaccination (25). Night and shift work is expected to alter immune responses to infectious agents as reported in field studies where hospital shift workers were more prone to display common, respiratory or COVID-19 infections (10, 16, 26, 27). In addition, night workers may be more prone to develop cancers due to the circadian clock disruption of T suppressor/cytotoxic and NK Cells, that play an important role in the detection and elimination of cancer cells (28). Circadian clocks have a major role to coordinate the expression profile of immune defences ensured by leucocyte and cytokines for a fine-tuned inflammatory response potentially altered in shift workers.

Immune and inflammatory changes in shift workers remain a relatively recent research field (22). However, several investigations mostly based on a single morning blood sample during a medical examination associated shift work with changes in leucocytes subtypes counts or activity (neutrophils, lymphocytes, NK Cells) and in inflammatory cytokines and C-reactive protein (CRP) (29–32). CRP, a hepatic protein of the acute inflammation phase is stimulated by pro-inflammatory cytokines such as interleukin-8 (IL-8) or interleukin-6 (IL-6). Under normal physiological conditions, there are low concentrations of cytokines in the blood, except for IL-6, a cytokine with a biphasic circadian secretion pattern and peak levels reported to occur in the morning and in the early evening in healthy women and men (33, 34).

The consequences of circadian misalignment and sleep debt for inflammatory and immuno-hematologic variables need to be further investigated in night and shift workers using the most appropriate objective and dynamic approaches.

Most previous studies in shift workers have assessed the duration of the main sleep episode through a single question, and seldom took into account all the sleep episodes that could occur over the 24-h period. Here, we investigated both the duration of the main sleep episode, and the 24h total sleep time (TST) captured during a full workweek to take into account the role of napping in sleep debt status. Such a 24h-TST paradigm seems to us more appropriate to capture the habitually biphasic sleep of shift workers (35). Hospital workers (nurses and nurse assistants) were our target population in the present study as they represent the largest workforce category exposed to night work, including ~250,000 nurses and ~200,000 nurse assistants in France (36). Our aims were (i) to study the link between shift working rhythms and the immune responses assessed using a set of biomarkers, and (ii) to investigate the role of sleep patterns, including TST per 24 h, sleep debt and social jet-lag (a behavioral proxy of circadian misalignment) as possible determinants of the immune response.

## Methods

### Participants and setting

Participants were recruited among health professionals (nurses and assistant nurses) working in a Hospital from the Assistance Publique - Hôpitaux de Paris (AP-HP), the main public hospital institution in the Paris area. Recruitment was promoted through protocol presentations by the study investigators during meetings that took place in each department within the Hospital between November 2016 and November 2017. Study participants were working on rotating day shifts, i.e. either in the morning (7:00 - 14:00) or in the afternoon (14:00 - 21:00), or on permanent night shifts (21:00 - 7:00). We originally measured during a seven-day period the sleep debt (difference of TST per 24 h between free days and work days) and social jet-lag (difference between the average mid-sleep point from the main sleep episode on free days and that on work days), in association with the immune cell numbers and inflammatory biomarkers levels in the ecological environment of a large sample of hospital workers.

### Questionnaire

Each participant completed a self-administered questionnaire. We collected information on socio-demographic characteristics, lifestyle-related factors (tobacco and alcohol consumption), anthropometric characteristics, and lifetime occupational history. Chronotype was determined using the French version of the morningness-eveningness questionnaire (37, 38), and job strain was assessed using the French version of the “Job Content Questionnaire” established by Karasek (39). Medical conditions (cardiovascular, metabolic, digestive, renal, neurological, respiratory, musculoskeletal or neoplastic diseases) were assessed from the question “Do you have any of these diseases diagnosed by a doctor?”.

### Sleep and work diaries

During seven consecutive days on average (six to ten days), hospital workers reported every sleep event including naps on a 24-h diary, adapted from the consensus sleep diary (40). Each participant recorded the following information: (1) the time of going to bed; (2) the time of attempting to fall asleep; (3) the sleep onset latency; (4) the number of awakenings during sleep; (5) the duration of awakenings; (6) the time of final awakening: (7) the time of getting-up; (8) the time and duration of naps. A work diary combined with the sleep diary was completed to report the work hours over the 7 consecutive days. During the week of data collection, rotating day shifters (DS) may have worked during either morning shifts, afternoon shifts, or both, while night shifters (NS) have worked exclusively on night shifts.

### Collection of blood samples

**Figure 1:**
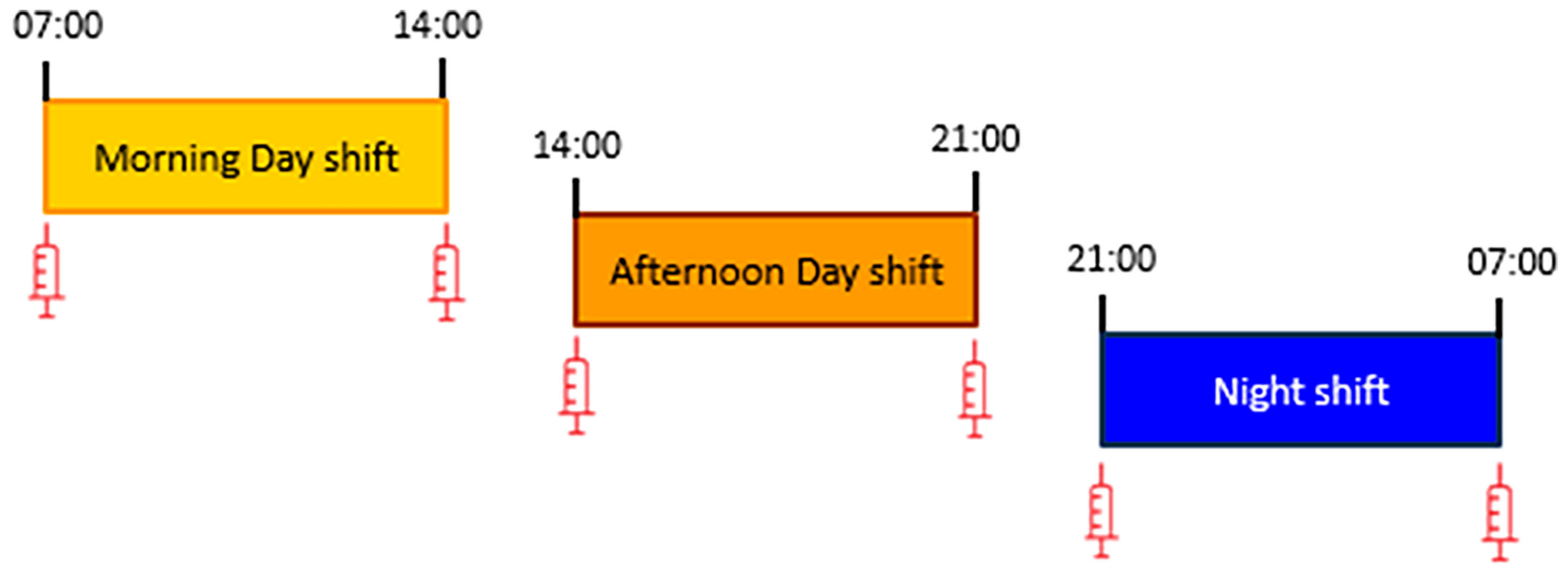
Schedules for blood sampling at the beginning and end of the work shift for morning (7:00 and 14:00), afternoon (14:00 and 21:00) and night (21:00 and 7:00) shifts.

Two blood samples were drawn at the beginning and end of the last shift of the 7 days of data collection. **Figure 1** shows the timing of blood sampling by shift type: day shifters whose last shift was a morning shift (MS) had blood drawn at 7:00 and 14:00; day shifters whose last shift was an afternoon shift (AS) had blood drawn at 14:00 and 21:00. All night workers (NS) had blood drawn at 21:00 and at 7:00.

### Total sleep time, sleep debt and social jet-lag

Total sleep time per 24 hours was calculated from the sleep diary as the duration of the main sleep episode (time from sleep onset to final awakening minus time spent awake) plus the duration of daily naps, averaged over the week. (TST24) was also assessed separately for work days (TST24w) and free days (TST24f).

In the analyses, the sleep patterns of study subjects can be fully characterized using the following parameters: total sleep time during work days (TST24w), sleep debt defined as TST24f – TST24w (41), and social jet-lag, a behavioral proxy of circadian misalignment defined as the difference between the average mid-sleep point from the main sleep episode on free days and that on workdays (42). The median sleep time was derived from the center time between the falling asleep time and the last awakening time (42).

### Leukocyte counts and subsets, NK Cells, CRP and interleukin-6 assays

Blood samples (10 mL) were obtained from an antecubital vein using a needle stick at the beginning and the end of a shift: at 07:00 and 14:00 for morning shift, at 14:00 and 21:00 for afternoon shift and at 21:00 and 07:00 for night shift (**Figure 1**). Plasma samples were harvested in citrated vacuum tubes and whole blood was collected in EDTA-treated tubes. Plasma samples were assayed using commercially available ELISA kits with high-sensitivity for CRP and IL6 determinations (R&D Systems, Minneapolis, MN, USA). The lower limits of ELISA detection for CRP and IL-6 concentrations were 0.059ng/ml and 0.01 ng/ml respectively. Average intra-assay coefficients of variation were 4.4% and 6.9% respectively. Corresponding average interassay coefficients of variation were 6.0 and 9.6%, respectively. Leukocyte counts and subsets were determined in EDTA-treated blood using an automated hemocytometer. Lymphocyte subset counts were determined following flow cytometry analysis of cell surface markers for CD3+ (all T-cells), CD4+ (T-Helper cells), CD8+ (Cytotoxic T-cells), CD19+ (B-cells) and CD16+/56+ (NK Cells) using a FACSCalibur (BD Biosciences, New Jersey, USA).

### Statistics

The log-transformed concentrations of CRP and IL6 as well as the leukocytes counts were compared between MS, AS and NS groups considered in pairs. We compared the mean values obtained from blood samples taken at the same time of the 24 h period, i.e., comparing the MS and NS groups at 7:00 h, the MS and AS groups at 14:00 h, and the AS and NS groups at 21:00 h (**Figure 1**). We also conducted multivariate linear regression models separately in MS, AS and NS groups. In these models the mean value of the biomarker between the two blood draws was taken as the dependent variable, and TST24w, sleep debt, and social jetlag were introduced as independent explanatory variables. The β regression parameters and their 95% confidence intervals of the sleep parameters were estimated after adjustment for age, body mass index (BMI), chronotype (morning, evening or neutral), tobacco status (current smoker, former smoker, non-smoker). All statistical analyses were conducted using SAS software version 9.4 and graphs were designed using RStudio software version 3.6.1

### Ethics

This study was reviewed and approved by the Comité de Protection des Personnes (CPP) Ile de France 1 on May 9, 2016, and by the Commission Nationale Informatique et Libertés (CNIL) in January 2017. All the participants provided their written informed consent prior to inclusion into the study. The study was carried out in accordance with the standards set by the latest revision of the Declaration of Helsinki.

## Results

### Socio-demographic characteristics by type of shift

Participants included 95 health professionals working permanent night shifts (NS) and 96 health professionals working rotating day shifts. Of these, 39 had blood drawn at the beginning and end of a morning shift (MS), and 57 had blood drawn at the beginning and end of an afternoon shift (AS). The 191 participants were mostly women (93.5%) and worked as nurses (50%), nurses’ assistants (45.5%) or health executives (4.5%). **Table 1** shows that, as compared to day shift (MS and AS) participants, NS workers were older (mean age 43.5 ± 10.5 vs. 38.6 ± 11.1 and 35.7 ± 10.3 in MS and AS, respectively) and had a higher proportion of overweight individuals, had longer work shifts (10 hours vs 7.7 h and 7.6 hours in MS and AS), had worked at night for more years (9.1 years vs 4.5 and 4.0 years in MS and AS), were more often of the evening chronotype (27% *vs* 8% and 12% in MS and AS). Commuting time to work was not different between NS and day shifters. Job strain and decision latitude (two different dimensions estimated from the Karasek questionnaire) showed that NS were less stressed and active and more passive and relaxed, as compared to MS and AS (*p* =0.0008). Tobacco, coffee or alcohol consumptions were similar in MS, AS and NS groups (**Table 1**).

**Table 1:** Selected characteristics of hospital workers by type of work shift.

	Morning shift		Afternoon shift		Night shift	
	(n=39)		(n=57)		(n=95)	
**Socio-demographic characteristics**						
**Age**						
*mean (SD)*	*38.6*	*11.1*	*35.7*	*10.3*	*43.5*	*10.5*
**Sex**						
Women	37	95%	55	96%	89	94%
Men	2	5%	2	4%	6	6%
**Partner**						
No	16	41%	16	28%	40	42%
Yes	23	59%	41	72%	55	58%
**Number of children at home**						
0	12	31%	23	40%	28	29%
1	13	33%	15	26%	23	24%
≥ 2	14	36%	19	33%	44	46%
**Characteristics of work**						
**Position**						
Care assistant	20	51%	27	47%	42	44%
Nurse	5	13%	0	0%	51	54%
Health executive	14	36%	30	53%	2	2%
**Number of years in current position**						
*mean (SD)*	*2.1*	*3.0*	*2.1*	*3.7*	*8.2*	*8.3*
**Number of hours worked per week**						
*mean (SD)*	*36.7*	*4.7*	*34.4*	*2.1*	*32.7*	*1.0*
**Duration of travel time**						
*mean (SD)*	*0.5*	*0.4*	*0.5*	*0.3*	*0.6*	*0.5*
**Night work in the entire work history**						
Never	26	67%	41	72%	0	0%
Ever	13	33%	16	28%	95	100%
**Number of years in night work for ever night worker**						
*mean (SD)*	*4.5*	*4.5*	*4.0*	*4.8*	*9.1*	*8.7*
**Time since quiting night work**						
*mean (SD)*	*9.1*	*8.7*	*9.3*	*8.5*	*0.0*	*0.0*
**Lifestyle habits**						
**Alcohol**						
Never	30	77%	39	68%	59	62%
< 2 drinks/week	4	10%	4	7%	13	14%
≥ 2 drinks/week	5	13%	14	25%	23	24%
**Tobacco**						
Never smoker	27	69%	37	65%	55	58%
Former smoker	3	8%	5	9%	18	19%
Current smoker	9	23%	15	26%	22	23%
**Body Mass Index (kg/m²)**						
Normal (<25)	23	59%	37	65%	35	37%
Overweight (25–29)	7	18%	10	18%	38	40%
Obese(≥ 30)	9	23%	10	18%	22	23%
**Chronotype**						
Morning type	13	33%	18	32%	11	12%
Neutral type	23	59%	32	56%	57	60%
Evening type	3	8%	7	12%	26	27%
**Medical history - Job Strain**						
**Personal diseases**						
Cardio-vascular diseases	2	5%	1	2%	5	5%
Metabolic diseases	3	8%	10	18%	16	17%
Digestive and renal diseases	4	10%	7	12%	16	17%
Neurological diseases	10	26%	15	26%	21	22%
Respiratory diseases	4	10%	5	9%	17	18%
Musculoskeletal diseases	1	3%	6	11%	6	6%
Cancers	0	0%	0	0%	2	2%
**Karasek (Job Strain)**						
Active	15	38%	18	32%	14	15%
Relaxed	6	15%	6	11%	28	29%
Passive	4	10%	7	12%	25	26%
Stressed	14	36%	26	46%	28	29%

Socio-demographic, work, lifestyle and health characteristics according to the type of shift.

### Total sleep time, sleep debt and social jetlag by type of shift

The average of the total sleep time per 24 h on work days (TST24w) was lower in NS (5.4 ± 1.5 h) than in rotating day shifters (6.4 h and 7.1 h in MS and AS, respectively). NS partly compensated for their lower sleep duration during work days by taking longer or/and more frequent naps during work days (1.7 ± 0.7 h in NS vs. 1.2 ± 0.6 h in MS and 1.3 ± 0.7 h in AS, *p* = 0.01 and 0.04 respectively, not shown), and had a slightly but not significantly higher total sleep time per 24 h on free days (TST24f = 8.5 ± 1.5 h in NS vs. 8.2 ± 1.1 h in MS and 8.1 ± 1.4 h in AS, *p* = 0.3 and 0.07 respectively). Nevertheless, the sleep debt (TST24f — TST24w) in NS was 3.2 ± 1.9 hours and remained significantly higher than in MS (1.9 ± 1.4 hours) and in AS (1.0 ± 1.5 h) (**Table 2**). The social jetlag was 1.75 ± 0.98 h in MS (calculated as the difference between median sleep time 2:06 on work days and median sleep time 4:18 on free days), 1.22 ± 0.79 h in AS (median sleep time 3:54 on work days and 4:18 on free days), and 6.69 ± 2.36 h in NS (median sleep time 11:48 on work days and median sleep time 4:30 on free days) in NS (**Table 2** and **Supplementary Figure S1**).

**Table 2:** Total sleep time, sleep debt and social jet-lag by type of shift and two-by-two comparisons of means.

	Morning shift	Afternoon shift	Night shift	*p-value MS vs NS*	*p-value MS vs AS*	*p-value NS vs AS*
	(n=39)	(n=57)	(n=95)			
**Total sleep time on work days (h)** Mean (SD)	6.35 (1.03)	7.08 (1.24)	5.37 (1.45)	*0.0002*	*0.0034*	*<0.0001*
**Total sleep time on free days (h)** Mean (SD)	8.28 (1.18)	8.12 (1.41)	8.57 (1.56)	*0.3012*	*0.568*	*0.0772*
**Sleep debt (h)** Mean (SD)	1.92 (1.46)	1.04 (1.50)	3.23 (1.94)	*0.0003*	*0.0056*	*<0.0001*
**Social jet-lag (h)** Mean (SD)	1.75 (0.98)	1.22 (0.79)	6.69 (2.36)	*<0.0001*	*0.0047*	*<0.0001*
Median sleep time on work days* Mean (SD)	2.82 (0.93)	3.29 (0.92)	11.72 (1.72)	*<0.0001*	*0.0158*	*<0.0001*
Median sleep time on free days* Mean (SD)	4.36 (1.09)	4.39 (1.22)	5.05 (1.92)	*0.0373*	*0.9045*	*0.0199*

*Time in decimal format. Mean values of Total Sleep Time per 24 h on work days (TST24w) was shorter in night shifters (NS) as compared to morning (MS) and afternoon shifters (AS) (all p < 0.0001). Sleep debt (TST per 24 h on free days – TST per 24 h on working days) and social jet lag were greater in night shift as compared to morning and afternoon shifters.

### Variations of immune and inflammatory biomarkers before and after a workshift

**Table 3** shows cell counts and IL6 and CRP concentrations at 7:00, 14:00 and 21:00 by shift group. At 7:00, lymphocytes, T-Cells, T-Helper Cells, cytotoxic T-Cells, B-Cells and IL6 were significantly higher in NS than in MS. All biomarkers measured at 14:00 had similar values in days shifters MS and AS. At 21:00, the lymphocyte counts including T-Cells, cytotoxic-T-Cells, NK Cells and IL6 were lower in NS than in AS.

**Table 3:** Age-adjusted means and standard deviation (SD) of immune cells and inflammatory biomarkers before and after workshift in morning, afternoon, and night shifters.

	Morning shift (n=39)	Afternoon shift (n=57)	Night shift (n=95)	p
	Mean *(SD)*	Mean *(SD)*	Mean *(SD)*	
**Lymphocytes (Cell/nL)**				
7:00	2.18 *(0.57)*		3.17 *(1.07)*	***<.0001***
14:00	2.41 *(0.57)*	2.23 *(0.64)*		*0.11*
21:00		2.89 *(0.74)*	2.73 *(0.9)*	*0.26*
**T- Cells (CD3) (Cell/µL)**				
7:00	1655 (423)		2398 *(804)*	***<.0001***
14:00	1825 *(449)*	1664 *(521)*		*0.09*
21:00		2207 *(599)*	2017 *(708)*	*0.09*
**T-Helper Cells (CD3-4) (Cell/µL)**				
7:00	1068 *(332)*		1623 *(556)*	***<.0001***
14:00	1171 *(360)*	1061 *(368)*		*0.11*
21:00		1424 *(438)*	1344 *(464)*	*0.30*
**Cytotoxic T-Cells (CD3-8) (Cell/µL)**				
7:00	550 *(172)*		742 *(318)*	***0.001***
14:00	608 *(193)*	589 *(246)*		*0.67*
21:00		767 *(290)*	658 *(317)*	***0.04***
**B Cells (Cell/µL)**				
7:00	265 *(134)*		498 *(304)*	***<.0001***
14:00	307 *(140)*	278 *(140)*		*0.23*
21:00		391 *(177)*	396 *(218)*	*0.88*
**NK cells (Cell/µL)**				
7:00	232 *(124)*		237 *(126)*	*0.99*
14:00	234 *(113)*	258 *(113)*		*0.53*
21:00		232 *(93)*	278 *(138)*	***0.03***
**Neutrophils (Cell/nL)**				
7:00	3.18 *(1.15)*		3.30 *(1.4)*	*0.57*
14:00	3.80 *(1.01)*	4.08 *(1.33)*		*0.20*
21:00		4.10 *(1.19)*	3.62 *(1.53)*	***0.04***
**Monocytes (Cell/nL)**				
7:00	0.50 *(0.18)*		0.56 *(0.2)*	*0.10*
14:00	0.51 *(0.15)*	0.48 *(0.16)*		*0.35*
21:00		0.55 *(0.17)*	0.54 *(0.18)*	*0.63*
**IL6 (pg/ml)**				
7:00	1.29 *(0.85)*		2.21 *(1.38)*	***0.0002***
14:00	1.31 *(0.67)*	1.44 *(1.11)*		*0.50*
21:00		2.18 *(1.69)*	1.69 *(1.17)*	***0.04***
**CRP (pg/ml)**				
7:00	2393 *(3299)*		2311 *(2530)*	*0.68*
14:00	2502 *(3715)*	2749 *(4019)*		*0.76*
21:00		2801 *(4060)*	2359 *(2605)*	*0.42*

Pairwise comparisons of means at equal times of the day (p-values in bold indicate p<0.05).

**Figure 2:**
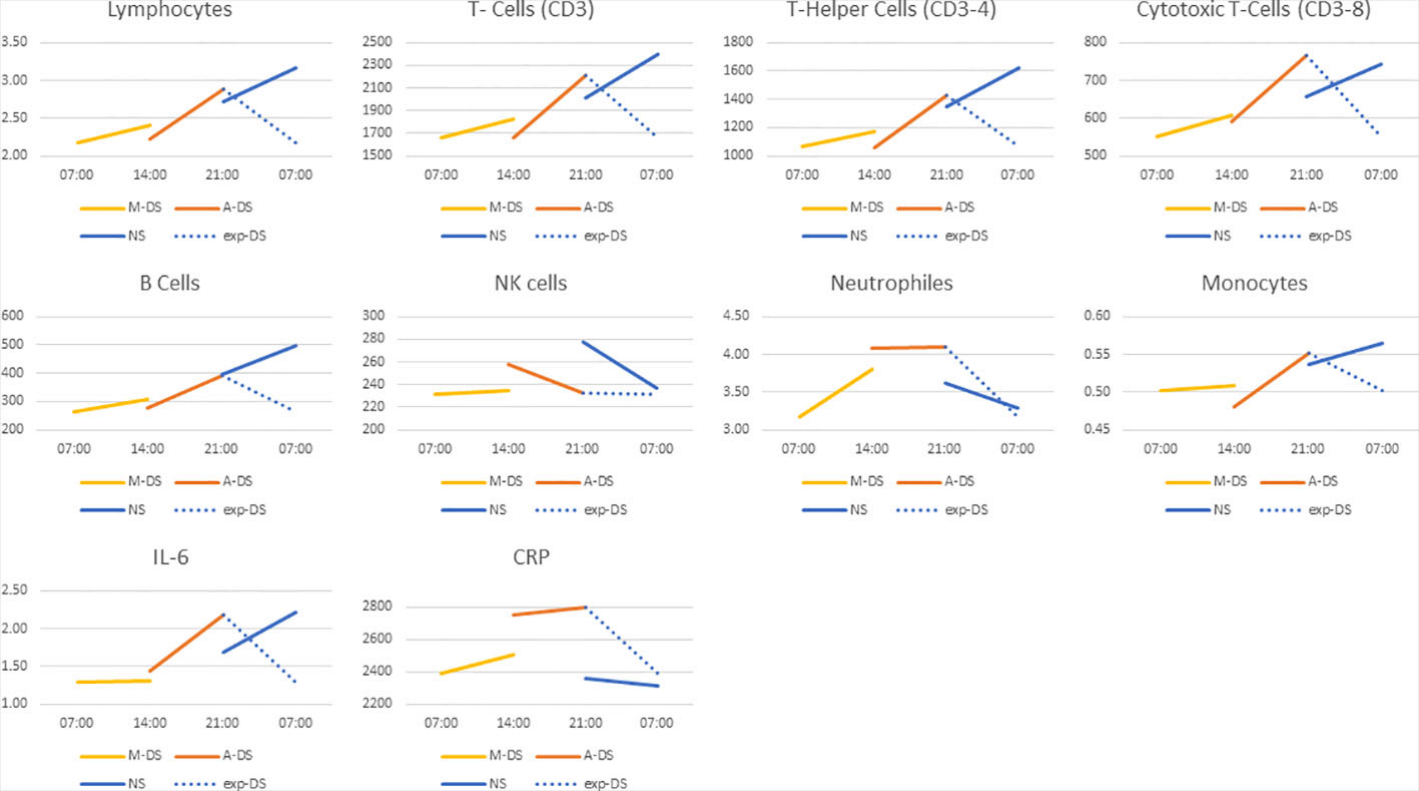
Mean values of biomarkers levels by shift group measured at start and end of the work shift (MS, Morning shift; AS, Afternoon shift; NS, Night shift; exp-DS, expected variation of biomarker level among day shifters between 21:00 and 7:00).

**Figure 2** shows the 24 hour variations of the biomarkers using the data collected in the 3 groups. In day shifters (yellow and orange lines for MS and AS), the levels of lymphocytes, including T-Cells, T-Helper Cells, T-Cytotoxic Cells, B-Cells as well as monocytes and IL6 were lowest at 7:00, increased during the day, and reached the highest levels at 21:00, consistent with the expected circadian variations for these biomarkers. The blue dotted line represents the expected decrease in biomarkers levels among day shifters during the night (not measured). Conversely, the variation in these biomarkers among NS (blue line) were lowest at 21:00 and highest at 7:00 at the end of the work shift, indicating strong phase shift of biomarkers levels in night workers. Changes in NK Cells, neutrophils are less clear. Interestingly, for the only assessed molecule, CRP without 24h variations, such a changed expression pattern was not observed. We have also checked across all participants if the day before the blood sampling was a work day or a free day, and found that the biomarkers values within each group (night shift, morning shift and afternoon shift) did not differ significantly depending on whether the previous day was a work day or a free day (all *p* > 0.05 between free or work day before the blood sampling) providing reassurance that this had only little impact on our results.

### Total sleep time, sleep debt, and social jet-lag effects on immune changes within morning, afternoon and night shift hospital workers

The role of sleep parameters (TST24w, sleep debt, social jetlag) on the levels of biomarkers can be examined in **Table 4** for each shift group separately. Among day shifters (MS and AS), all ß coefficients were close to zero, indicating that TST24w, sleep debt and social jetlag did not significantly affect biomarkers levels. Among NS, TST24w, sleep debt and social jetlag were significantly associated with total lymphocytes, and T-helper Cells (CD3-4). In addition, TST24w was positively associated with B-Cell and negatively with neutrophils, while social jetlag was associated with IL6 levels. These results indicate that the variation of immune biomarkers levels among NS are influenced by sleep parameters.

Because of the correlations between TST24w, sleep debt and social jetlag, the β coefficients associated with each parameter in **Table 4** should not be interpreted as a measure of the specific effect of each of them, but rather as a global effect of the circadian misalignment of night workers on the immune markers.

**Table 4:** Multivariate models showing the effects on immune biomarkers of total sleep time during work days (TST24w), sleep debt and social jet-lag among morning, afternoon and night shifters.

	Morning shift		Afternoon shift		Night shift	
	β	*p*	β	*p*	β	*p*
**Lymphocytes (Cell/nL)**						
Total sleep time on work days	-0.070	*0.50*	0.044	*0.64*	0.214	***0.03***
Sleep debt	-0.078	*0.29*	0.034	*0.69*	0.176	***0.01***
Social jet-lag	-0.050	*0.50*	-0.003	*0.98*	0.106	***0.02***
**T- Cells (CD3) (Cell/µL)**						
Total sleep time on work days	-0.053	*0.54*	0.029	*0.71*	0.134	*0.08*
Sleep debt	-0.044	*0.47*	0.031	*0.66*	0.142	***0.01***
Social jet-lag	-0.034	*0.58*	-0.002	*0.99*	0.068	*0.05*
**T-Helper Cells (CD3-4) (Cell/µL)**						
Total sleep time on work days	-0.070	*0.34*	0.011	*0.84*	0.108	***0.03***
Sleep debt	-0.039	*0.45*	0.040	*0.40*	0.108	***0.002***
Social jet-lag	-0.004	*0.94*	-0.036	*0.66*	0.050	***0.03***
**Cytotoxic T-Cells (CD3-8) (Cell/µL)**						
Total sleep time on work days	-0.006	*0.90*	0.006	*0.87*	0.023	*0.49*
Sleep debt	-0.019	*0.53*	-0.006	*0.85*	0.031	*0.19*
Social jet-lag	-0.034	*0.28*	0.042	*0.46*	0.020	*0.21*
**B Cells (Cell/µL)**						
Total sleep time on work days	-0.002	*0.93*	-0.008	*0.71*	0.061	***0.03***
Sleep debt	0.013	*0.54*	-0.002	*0.92*	0.023	*0.22*
Social jet-lag	0.015	*0.48*	0.003	*0.92*	0.022	*0.09*
**NK cells (Cell/µL)**						
Total sleep time on work days	0.010	*0.98*	0.065	*0.68*	0.212	*0.15*
Sleep debt	-0.057	*0.80*	0.056	*0.70*	0.110	*0.28*
Social jet-lag	-0.159	*0.50*	-0.231	*0.34*	0.125	*0.07*
**Neutrophils (Cell/nL)**						
Total sleep time on work days	-0.033	*0.89*	0.267	*0.07*	-0.307	***0.03***
Sleep debt	0.086	*0.60*	-0.058	*0.66*	-0.140	*0.15*
Social jet-lag	-0.375	***0.03***	0.143	*0.52*	-0.051	*0.43*
**Monocytes (Cell/nL)**						
Total sleep time on work days	0.008	*0.76*	-0.001	*0.96*	-0.012	*0.53*
Sleep debt	-0.001	*0.95*	0.010	*0.62*	-0.013	*0.31*
Social jet-lag	-0.026	*0.18*	0.005	*0.89*	0.002	*0.77*
IL6* (pg/ml)						
Total sleep time on work days	0.017	*0.82*	0.019	*0.81*	-0.020	*0.68*
Sleep debt	0.070	*0.26*	0.031	*0.67*	-0.023	*0.49*
Social jet-lag	-0.030	*0.72*	-0.070	*0.56*	0.052	***0.03***
CRP ** (pg/ml)						
Total sleep time on work days	0.010	*0.98*	0.087	*0.54*	-0.151	*0.17*
Sleep debt	-0.029	*0.90*	0.235	*0.06*	-0.086	*0.26*
Social jet-lag	0.164	*0.46*	-0.254	*0.21*	-0.035	*0.50*

*IL6 values were log transformed in the regression model. **CRP values were divided by 1000 and log-transformed in the regression model. The β coefficients represent the variation of the biomarker concentration for each additional hour of TST24w, sleep debt and social jetlag. All models are adjusted for age, body mass index, chronotype (morning, evening, neutral) and smoking status (current smoker, former smoker, never smoker). p-values in bold indicate p≤0.05.

## Discussion

Our study found that night shift condition was associated with altered pattern expressions of immune cells and a pro-inflammatory cytokine that display diurnal rhythms. While variations of most immune biomarkers concentrations during morning and afternoon shifts were consistent with the expected diurnal variations among day workers, IL6, all lymphocyte subtypes tested, neutrophils, NK and B Cells showed a strong circadian phase shift among night workers with peak values observed in the morning instead of the evening.

### Immune markers and work time schedule

The usual expression profile in diurnal healthy subjects for lymphocyte subsets and neutrophil shows peaks late evening 23:00 and afternoon respectively and nadir values in mid- and early morning respectively (17). In our study, the 24 h variations of immune cells and IL6 were not significantly affected in rotating day shifters. Although, the rotating day nurses were exposed alternately to morning and afternoon shifts, the level of immune markers was similar at 14:00 in day shifters ending their morning or starting their afternoon shift. This suggests to certain extent a homogeneity in the responses of the immune system to the work schedules in the group of rotating day nurses and no main influence of having worked or not before the blood collection during the daytime period. By contrast, hospital workers on permanent night shifts are expected to have a different entrainment of circadian rhythms than those on rotating day shift, due to their different sleep-wake rhythms and periods of light exposure during a 24-h day. Accordingly, the present immune modifications in NS most likely results from a phase shift of the normal circadian immune rhythm measured here for immune biomarkers sensitive to social jet-lag, i.e. IL6 and T-Helper cells. Measured changes in these immune parameters could reflect a phase variation of the circadian pacemaker brought about by peripheral blood mononuclear cell gene expression (32), but some immune changes were also explained by their sensitivity to sleep duration or debt (T-Helper cells, neutrophil or B Cells).

Neutrophil and lymphocyte subset levels have been well documented to be sensitive to the rates of sleep debt in experimental sleep deprivation investigations (14, 17). Specifically, neutrophil counts could be an immune sensor of the level of sleep duration in the light of our previous data (43, 44) consistently with the present analyses (each additional hour of sleep decreases by 0.31 neutrophil counts in NS).

Sleep debt and social jet-lag (a behavioral *proxy* of circadian misalignment) can influence immune responses. However, these two processes have complex and intimate interactions that are hard to discriminate. We are also aware that night and day workers may differ in many other aspects in addition to levels of social jet lag and sleep debt (i.e. timing of light exposure, meal timing, activity patterns, chronotype, sociodemographics, lifestyle) but several relevant adjustments were performed here.

### Sleep debt

Sleep debt was measured per 24h, and not considering only the main sleep episode. Such a 24h paradigm seems to us more appropriate to capture the habitual biphasic sleep of shift workers, since both night and morning shifts shorten the main sleep episode, reduce the amount of Rapid Eye Movement (REM) sleep and stage 2 sleep, and are associated with taking naps in the afternoon (35). Scarcely studied, the few reports on TST per 24h showed that 30% to 46% hospital workers reported sleeping less than 6 h/24 h (45, 46). We reported a mean sleep debt of 3.1 h in NS while it was two times smaller in rotating MS and AS (1.5 h in average). Transportation time between home and work place were comparable in NS, MS and AS and cannot account for the sleep debt difference.

### Social jet-lag

Regarding now the desynchronization of the biological clock from sleep-wake and working rhythms cues, rapid shift rotations are mainly associated with a damping of the rhythm amplitude while in fixed night shift workers part of the subjects shift more or less spontaneously melatonin secretion towards the period of morning sleep (47). In a similar range to Teixeira *et al.* who reported a social jet lag of 5.1 [2.6-7.9] h in night workers compared to 1.2 [0.7-2.0] h in day worker (48), we found a social jet-lag of 6.8 [5.2-8.2] h in NS and only 0.9 [0.0-2.2] h and 1.7 [1.0-2.4] h in AS and MS respectively. The impact of this circadian disruption has been investigated in experimental circadian misalignment mimicking night shift work on a short period. Data showed that it induced a loss of rhythmicity and/or dampened/blunted rhythms as well as overexpression of immune markers (18, 20, 21). Here, the changes measured in immune biomarkers could reflect that the rhythm has shifted to peak at the same circadian phase but a different clock time across individuals. The statistical models used here (i.e. testing the effects of social jet-lag, sleep debt and total sleep time on immune changes after multiple adjustments) are in part in favor of such an hypothesis but also combined with a partial sleep debt effect.

### What we previously knew on inflammatory responses to shift work setting

In the present study, the profile of IL6 levels was markedly changed in NS. In addition, the IL6 concentrations in day shift workers were in the same range as those observed in healthy subjects for whom usual IL6 levels assessed from serial blood samplings display an acrophase at ~21:00 h and a nadir at ~7:00-8:00 (33, 34). Of note, changes in the inflammatory system are observed here in a biomarker that displays a pseudo-circadian expression (i.e. IL6) and not for a biomarker without circadian regulation [i.e. CRP (49)]. This likely phase shift of 24h-IL6 variation could not be easily captured in previous night work studies, since they used only a single morning blood sampling point and concluded to an IL6 overexpression rather than a shift of the circadian rhythms (here we reported as well an almost twice IL6 levels for the 7:00 morning point). We chose to measure IL6 and CRP by making the assumption that CRP, which is stable over 24h, will be mainly sensitive to sleep debt rather than social jet-lag, while IL6 will be sensitive to both altered processes i.e. sleep debt and circadian disruption. Nevertheless, no such discriminating effect regarding CRP came out from our analyses. In a more sophisticated way, some authors used a simulated night shift protocols but included shift workers and measured elevated CRP levels every 4 hours for high sensitivity CRP assessment with intravenous cannula. These multiple blood samples must be considered with caution since the cannula per se has been reported to increase local inflammation (e.g. IL6 (50),) which could be misleading since relatively small CRP variations are expected in this kind of protocol. However, elevated 24-h hs-CRP levels were measured during circadian misalignment protocol and were increased by 11% (20). But in shift work occupational settings, several reports used one single blood sampling in the morning after extended work shift in medical doctors or in a large cohort of airline employees and found higher CRP values after controlling for numerous potential confounding factors (29, 51). However, the NHS II (Nurses’ Health Study II) cohort showed no association between recent night shifts and CRP levels (CRP levels were non significantly higher among nurses who had worked rotating night shifts for more than one year compared to nurses who had never worked rotating night shifts). They only used one time-point of blood collection and determined with questionnaires the number of night shifts worked in the 2 weeks before the blood sampling, and total years of rotating night shift work (52).

### Potential clinical relevance for health professionals on night shift

There is increasing evidence that the desynchronization between the biological clock and working rhythms contributed to the pathogenesis of several medical conditions in shift workers (53). Immuno-inflammatory deregulation in nurses working night shifts could be one of the first steps leading to cardiovascular and tumour pathogenesis (14). Indeed, controlled laboratory investigations links circadian misalignment but also sleep deprivation to deregulation of the immune-inflammatory, metabolic and cardiovascular functions (54, 55). Epidemiological data suggest that shorter sleep duration increases the cardiovascular risk through increased incidence of diabetes, obesity or hypertension (56–58). It has been recently reported that compared to day workers, night shift workers - with lower sleep duration during both working and free days and higher social jet lag - displayed 3 times higher risk of abdominal obesity independent of age and gender (59). Also, sleeping at the wrong circadian times - and consecutive reduced sleep duration and quality - are associated to disruptions of the circadian rhythm of the immune innate and adaptive functions and does not help to combat infectious diseases. When investigating the non-circadian CRP inflammatory marker, no difference was detected between shift groups. This could advocate that (i) we are in absence of an acute phase response of inflammation during which IL6 stimulates the release of CRP by the liver; (ii) the circadian immune modulator IL6 is impacted and is possibly less able to respond to an immune challenge. CRP and IL6 play both a major role in the acute response of inflammation but IL6 has also both pro and anti-inflammatory properties (ability to inhibit pro-inflammatory cytokines) (60, 61). Pro-inflammatory cytokines such as IL6 have specific receptors that regulate the balance of the inflammatory response. This contributes to coordinate the acute phase of inflammatory response to a potential stressor with a relevant immune homeostasis. Disrupted pattern of expression of these immune mediators in night shift workers submitted to chronic stress - i.e. circadian desynchronisation and extended wakefulness - could have critical health implications (62, 63).

Also, T-Helper cells along with Cytotoxic-T cells represent the majority of T-lymphocytes and displayed both different cell counts in the time points measured here in night workers as compared to day workers.

Such disrupted pattern of expression reported here and similarly in a previous shift work study [i.e. higher morning T-cells counts in NS (64)] could impair the immune response to infection. Shift workers, especially those working the night shift, had a higher risk of infections, such as colds, flu and gastroenteritis, compared to those working daytime shifts (26). An important function of the rhythmicity of the immune system is to coordinate the different immune functions with each other. Considering the critical functions of T-cells in clearing infection following entry into the body, the temporal changed pattern expression of the T-cells could increase the vulnerability to infectious agents since the regulation of immune responses depending of the time of the day is optimized to provide protection at specific times (16). In other words, this means that the efficiency of the immune responses to pathogens (which have their own intrinsic clock) changes with time of the day (i.e. circadian drive component) and that night workers with disrupted circadian rhythms may be more vulnerable to potential environmental infection during their night shift (23, 24). During a chronic stress condition, because of a change in hormonal production or a desensitization of their effects, some immune cells or/and their activity falls down and this potential immunodeficiency could as well impact the onset of new pathologies. This higher infection susceptibility has been confirmed from several kinds of data in sleep-deprived and stressful conditions: vaccine experimentation indicates that sleep-mediated factors play an important role in the formation of the immunological memory (65); subjects with shorter sleep durations were more vulnerable to rhinovirus or pneumonia (66, 67); and higher incidence and severity of respiratory infections has been reported in shift workers (10). Recently, it was also reported that in response to meningococcal C meningitis vaccine administered at 7:00 the morning and night workers had weaker antigen-specific IgG responses 28 and 56 days following vaccination. Lower prolactin rates (a key hormone SWS-dependant in the Ag-specific immune response) and reduced CD4-T lymphocytes (T-Helper cells) were also measured during this post-vaccination period. During the 4 weeks after vaccination in night workers, shorter TST was indeed associated with lower specific antibody levels suggesting that the humoral response to vaccination may be impaired in individuals with chronic sleep restriction and circadian misalignment (68).

### Limitations of the study

We tried here as far as possible to disentangle the respective roles of circadian disruption and sleep debt in the immune responses to shift working rhythms. Although we found that sleep debt combined or not with social-jet lag had an impact on immune biomarkers, it remains to consolidate and improve in the future the understanding of the respective roles of sleep debt, on one side, and circadian misalignment on the other side, because of complex interactions between circadian rhythms and sleep patterns. Although the added value of our design was to perform a second blood sampling, a more systematic registration of the full circadian profile would improve our understanding in relation to changes for immunological expressions. Rotating day and night workers have potentially distinct circadian patterns and the same time point may correspond to different phases of the circadian rhythm for immune biomarkers that display 24-h variations. Serial blood samplings would have been preferable but unrealistic without interfering with the nurse’s hospital work and to obtain their agreement for participating in the study. Also, multiple blood samplings could lead to bias measurements [multiple blood samples must be considered also with caution since as mentioned above the cannula *per se* has been reported to increase local inflammation, e.g. IL-6 (33)] and we performed two blood samplings before and after the working period. Distinct time intervals between blood sample time points was performed because of shift work schedules (10 hours in the NS group but 7 hours in the MS and AS groups). In addition, we did not control for fasting duration prior to each blood collection time point. Although to our knowledge, most of the immune biomarkers assessed here were not especially sensitive to the fasting condition, timing of food intake and fasting duration could affect insulin sensitivity and glucose but also inflammatory markers (69–72). Furthermore, we could hypothesize that the reported biological alterations would have certainly been even more noticeable in night workers if compared to a fixed day working nurses (e.g. standard daily working hours (07:00/08:00 - 17:00/18:00) rather than rotating day shifters that are submitted to alternated working rhythms. But the small number of hospital staff with this type of schedule did not allow us to carry out these analyses and we reported a homogeneity in the responses of the immune system to their work schedules in the group of rotating day nurses working morning or afternoon shifts.

Also, a potential bias for social-jet lag calculation could result from the fact that free days do not reflect perfectly the spontaneous pattern of the person, because they are compensating the poor sleep during shift work. Future studies should probably investigate how to precisely correct the social-jet lag for sleep debt. Social-jet lag remains however an interesting proxy of the level of stress on the circadian regulation of the immune system exerted by social timing constraints.

The same limitation could be addressed for the sleep debt calculation since during the free days the participants recover from the sleep debt accumulated on workdays and possibly oversleep as compared to their exact physiological need. Finally, the serial measurements of a central marker of the biological clock (central temperature or melatonin) could have helped to better understand the phase relations between the peripheral circadian immune rhythm and the central rhythm. However, circadian rhythmicity in activity and body surface temperature was telemonitored continuously on work and free days in the same nurses during the week of investigation. The obtained results suggest that the rest-activity rhythms of the night shifters were phase shifted as a group, with damped rest-activity pattern and suppressed chest temperature rhythms for the majority of them as compared to rotating day shifters (73).

## Conclusions

Altogether, our data point to intricate response patterns of immune-inflammatory rhythms to social jet-lag (a behavioral proxy of circadian misalignment) and sleep debt rates in night shifters. Specifically, this disrupted pattern expression of immune cells in hospital night workers could increase the vulnerability to infections and reduces vaccination efficiency in night workers.

## Data availability statement

The original contributions presented in the study are included in the article/**Supplementary Material**. Further inquiries can be directed to the corresponding author.

## Ethics statement

This study was reviewed and approved by the Comité de Protection des Personnes (CPP) Ile de France 1 on May 9, 2016, and by the Commission Nationale Informatique et Libertés (CNIL) in January 2017. The patients/participants provided their written informed consent to participate in this study.

## Author contributions

BF, FL, DL, and PG contributed to conception and design of the study. PG supervised the data collection. GA and CD performed the study and biological analyses, EC-D, GA, and CG organized the database. EC-D and PG performed the statistical analysis. BF wrote the first draft of the manuscript. All authors contributed to the article and approved the submitted version.

## Funding

The CIRCADIEM study was supported by a research grant of the PNREST programme 2016 from the French public agency ANSES (Agence nationale de sécurité sanitaire de l'alimentation, de l'environnement et du travail) and by the Institut National de la Santé et de la Recherche Médicale (File #C15-66, INSERM, France). The funders had no role in study design, data collection and analysis, decision to publish, or preparation of the manuscript.

## Acknowledgments

We would like to thank very sincerely the hospital workers for their participation in the study; and also the Direction des Ressources Humaines de l’AP-HP, Département Santé au Travail et Politique Sociale; Claire Mulot and the CRB Epigenetec, INSERM UMR-S 1147, Université Paris Descartes, the Laboratoire de biochimie-hématologie de l’Hôpital Paul-Brousse, Villejuif and the Laboratoire d’immunologie de l’Hôpital Bicêtre, Kremlin-Bicêtre.

## Conflict of interest

The authors declare that the research was conducted in the absence of any commercial or financial relationships that could be construed as a potential conflict of interest.

## Publisher’s note

All claims expressed in this article are solely those of the authors and do not necessarily represent those of their affiliated organizations, or those of the publisher, the editors and the reviewers. Any product that may be evaluated in this article, or claim that may be made by its manufacturer, is not guaranteed or endorsed by the publisher.

## Supplementary material

Supplementary Figure 1 Total sleep time, sleep debt and social jet-lag according to the type of shift. Total Sleep Time (TST) per 24 h on work days (based on the typical hours reported on sleep diary on work days), sleep debt (TST *per* 24 h on free days – TST *per* 24 h on working days) and social jet-lag status (difference between the average mid-sleep point from the main sleep episode on free days and that on workdays) are shown here as medians (25%–75% range).

## References

[1] IARC working group. Night Shift Work. In: IARC Monographs on the Identification of Carcinogenic Hazards to Humans, vol. 124. (2020). p. 52–4. Lyon, France:WHO IARC.

[2] Pilcher JJ Lambert BJ Huffcutt AI. Differential effects of permanent and rotating shifts on self-report sleep length: A meta-analytic review. Sleep (2000) 23:155–63. doi: 10.1093/sleep/23.2.1b 10737332

[3] Ohayon MM Smolensky MH Roth T. Consequences of shiftworking on sleep duration, sleepiness, and sleep attacks. Chronobiol Int (2010) 27:575–89. doi: 10.3109/07420521003749956 20524802

[4] Ganesan S Magee M Stone JE Mulhall MD Collins A Howard ME. The impact of shift work on sleep, alertness and performance in healthcare workers. Sci Rep (2019) 9:4635. doi: 10.1038/s41598-019-40914-x 30874565 PMC6420632

[5] Pan A Schernhammer ES Sun Q Hu FB. Rotating night shift work and risk of type 2 diabetes: Two prospective cohort studies in women. PloS Med (2011) 8:e1001141. doi: 10.1371/journal.pmed.1001141 22162955 PMC3232220

[6] Torquati L Mielke GI Brown WJ Kolbe-Alexander T. Shift work and the risk of cardiovascular disease. A systematic review and meta-analysis including dose-response relationship. scand. J Work Environ Health (2018) 44:229–38. doi: 10.5271/sjweh.3700 29247501

[7] Vyas MV Garg AX Iansavichus AV Costella J Donner A Laugsand LE. Shift work and vascular events: Systematic review and meta-analysis. BMJ (2012) 345:e4800. doi: 10.1136/bmj.e4800 22835925 PMC3406223

[8] Wegrzyn LR Tamimi RM Rosner BA Brown SB Stevens RG Eliassen AH. Rotating night-shift work and the risk of breast cancer in the nurses' health studies. Am J Epidemiol (2017) 186:532–40. doi: 10.1093/aje/kwx140 PMC5856106 28541391

[9] Cordina-Duverger E Menegaux F Popa A Rabstein S Harth V Pesch B. Night shift work and breast cancer: A pooled analysis of population-based case-control studies with complete work history. Eur J Epidemiol (2018) 33:369–79. doi: 10.1007/s10654-018-0368-x 29464445

[10] Loef B van Baarle D van der Beek AJ Sanders EAM Bruijning-Verhagen P Proper KI. Shift work and respiratory infections in healthcare workers. Am J Epidemiol (2019) 188:509–17. doi: 10.1093/aje/kwy258 PMC6395171 30475977

[11] Depner CM Melanson EL Eckel RH Snell-Bergeon JK Perreault L Bergman BC. Ad libitum weekend recovery sleep fails to prevent metabolic dysregulation during a repeating pattern of insufficient sleep and weekend recovery sleep. Curr Biol (2019) 29:957–67. doi: 10.1016/j.cub.2019.01.069 PMC12798825 30827911

[12] Simpson NS Diolombi M Scott-Sutherland J Yang H Bhatt V Gautam S. Repeating patterns of sleep restriction and recovery: Do we get used to it? Brain Behav Immun (2016) 58:142–51. doi: 10.1016/j.bbi.2016.06.001 PMC5067189 27263430

[13] Simpson NS Scott-Sutherland J Gautam S Sethna N Haack M. Chronic exposure to insufficient sleep alters processes of pain habituation and sensitization. Pain (2018) 159:33–40. doi: 10.1097/j.pain.0000000000001053 28891869 PMC5832516

[14] Besedovsky L Lange T Haack M. The sleep-immune crosstalk in health and disease. Physiol Rev (2019) 99:1325–80. doi: 10.1152/physrev.00010.2018 PMC6689741 30920354

[15] Ackermann K Revell VL Lao O Rombouts EJ Skene DJ Kayser M. Diurnal rhythms in blood cell populations and the effect of acute sleep deprivation in healthy young men. Sleep (2012) 35:933–40. doi: 10.5665/sleep.1954 PMC3369228 22754039

[16] Scheiermann C Gibbs J Ince L Loudon A. Clocking in to immunity. Nat Rev Immunol (2018) 18:423–37. doi: 10.1038/s41577-018-0008-4 29662121

[17] Lange T Dimitrov S Born J. Effects of sleep and circadian rhythm on the human immune system. Ann NY Acad Sci (2010) 1193:48–59. doi: 10.1111/j.1749-6632.2009.05300.x 20398008

[18] Kervezee L Cuesta M Cermakian N Boivin DB. Simulated night shift work induces circadian misalignment of the human peripheral blood mononuclear cell transcriptome. Proc Natl Acad Sci USA (2018) 115:5540–5. doi: 10.1073/pnas.1720719115 PMC6003514 29735673

[19] Kervezee L Kosmadopoulos A Boivin DB. Metabolic and cardiovascular consequences of shift work: The role of circadian disruption and sleep disturbances. Eur J Neurosci (2020) 51:396–412. doi: 10.1111/ejn.14216 30357975

[20] Morris CJ Purvis TE Mistretta J Hu K Scheer FAJ. Circadian misalignment increases c-reactive protein and blood pressure in chronic shift workers. J Biol Rhythms (2017) 32:154–64. doi: 10.1177/0748730417697537 PMC5858578 28347188

[21] Cuesta M Boudreau P Dubeau-Laramée G Cermakian N Boivin DB. Simulated night shift disrupts circadian rhythms of immune functions in humans. J immunol (2016) 196:2466–75. doi: 10.4049/jimmunol.1502422 26873990

[22] Faraut B Bayon V Léger D. Neuroendocrine, immune and oxidative stress in shift workers. Sleep Med Rev (2013) 17:433–44. doi: 10.1016/j.smrv.2012.12.006 23618533

[23] Edgar RS Stangherlin A Nagy AD Nicoll MP Efstathiou S O'Neill JS. Cell autonomous regulation of herpes and influenza virus infection by the circadian clock. Proc Natl Acad Sci USA (2016) 113:10085–90. doi: 10.1073/pnas.1601895113 PMC5018795 27528682

[24] Deng W Zhu S Zeng L Liu J Kang R Yang M. The circadian clock controls immune checkpoint pathway in sepsis. Cell Rep (2018) 10:366–78. doi: 10.1016/j.celrep.2018.06.026 PMC6094382 29996098

[25] de Bree LCJ Mourits VP Koeken VA Moorlag SJ Janssen R Folkman L. Circadian rhythm influences induction of trained immunity by BCG vaccination. J Clin Invest (2020) 130:5603–17. doi: 10.1172/JCI133934 PMC7641012 32692732

[26] Mohren DC Jansen NW Kant IJ Galama J van den Brandt PA Swaen GM. Prevalence of common infections among employees in different work schedules. J Occup Environ Med (2002) 44:1003–11. doi: 10.1097/00043764-200211000-00005 12449906

[27] Maidstone R Anderson SG Ray DW Rutter MK Durrington HJ Blaikley JF. Shift work is associated with positive COVID-19 status in hospitalised patients. Thorax (2021) 76:601–6. doi: 10.1136/thoraxjnl-2020-216651 PMC8098298 33903187

[28] Logan RW Zhang C Murugan S O'Connell S Levitt D Rosenwasser AM. Chronic shift-lag alters the circadian clock of NK cells and promotes lung cancer growth in rats. J Immunol (2012) 188:2583–91. doi: 10.4049/jimmunol.1102715 PMC3294088 22308312

[29] Puttonen S Viitasalo K Härmä M. Effect of shiftwork on systemic markers of inflammation. Chronobiol Int (2011) 28:528–35. doi: 10.3109/07420528.2011.580869 21797781

[30] Nagai M Morikawa Y Kitaoka K Nakamura K Sakurai M Nishijo M. Effects of fatigue on immune function in nurses performing shift work. J Occup Health (2011) 53:312–9. doi: 10.1539/joh.10-0072-OA 21778660

[31] Wirth MD Andrew ME Burchfiel CM Burch JB Fekedulegn D Hartley TA. Association of shiftwork and immune cells among police officers from the buffalo cardio-metabolic occupational police stress study. Chronobiol Int (2017) 34:721–31. doi: 10.1080/07420528.2017.1316732 PMC6649676 28488901

[32] Sookoian S Gemma C Fernández Gianotti T Burgueño A Alvarez A González CD. Effects of rotating shift work on biomarkers of metabolic syndrome and inflammation. J Intern Med (2007) 261:285–92. doi: 10.1111/j.1365-2796.2007.01766.x 17305651

[33] Vgontzas AN Zoumakis E Bixler EO Lin HM Follett H Kales A. Adverse effects of modest sleep restriction on sleepiness, performance, and inflammatory cytokines. J Clin Endocrinol Metab (2004) 89:2119–26. doi: 10.1210/jc.2003-031562 15126529

[34] Vgontzas AN Bixler EO Lin HM Prolo P Trakada G Chrousos GP. IL-6 and its circadian secretion in humans. Neuroimmunomodulation (2005) 12:131–40. doi: 10.1159/000084844 15905620

[35] Åkerstedt T. Shift work and disturbed sleep/wakefulness. Occup Med (Lond) (2003) 53:89–94. doi: 10.1093/occmed/kqg046 12637592

[36] Cordina-Duverger E Houot M Tvardik N El Yamani M Pilorget C Guénel P. Prévalence du travail de nuit en France : Caractérisation à partir d’une matrice emplois-expositions // prevalence of night work in France: Characterization from a job-exposure matrix. BEH (2019) 8-9:168–74. Available at: http://beh.santepubliquefrance.fr/beh/2019/8-9/2019_8-9_3.html

[37] Taillard J Philip P Chastang JF Bioulac B. Validation of Horne and ostberg morningness-eveningness questionnaire in a middle-aged population of French workers. J Biol Rhythms (2004) 19:76–86. doi: 10.1177/0748730403259849 14964706

[38] Horne JA Östberg O. A self-assessment questionnaire to determine morningness-eveningness in human circadian rhythms. In J Chronobiol (1976) 4:97–110. 1027738

[39] Niedhammer I Ganem V Gendrey L Sandra David S Degioanni S. Propriétés psychométriques de la version française des échelles de la demande psychologique, de la latitude décisionnelle et du soutien social du « job content questionnaire » de karasek : Résultats de l'enquête nationale SUMER. Santé Publique (2006) 3:413–42. doi: 10.3917/spub.063.0413 17094683

[40] Carney CE Buysse DJ Ancoli-Israel S Edinger JD Krystal AD Lichstein KL. The consensus sleep diary: Standardizing prospective sleep self-monitoring. Sleep (2012) 35:287–302. doi: 10.5665/sleep.1642 22294820 PMC3250369

[41] Leger D Richard JB Collin O Sauvet F Faraut B. Napping and weekend catchup sleep do not fully compensate for high rates of sleep debt and short sleep at a population level (in a representative nationwide sample of 12,637 adults). Sleep Med (2020) 74:278–88. doi: 10.1016/j.sleep.2020.05.030 32866843

[42] Roenneberg T Allebrandt KV Merrow M Vetter C. Social jetlag and obesity. Curr Biol (2012) 22:939–43. doi: 10.1016/j.cub.2012.03.038 22578422

[43] Faraut B Boudjeltia KZ Dyzma M Rousseau A David E Stenuit P. Benefits of napping and an extended duration of recovery sleep on alertness and immune cells after acute sleep restriction. Brain Behav Immun (2011) 25:16–24. doi: 10.1016/j.bbi.2010.08.001 20699115

[44] Lange T Born J. The immune recovery function of sleep -tracked by neutrophil counts. Brain Behav Immun (2011) 25:14–5. doi: 10.1016/j.bbi.2010.08.008 20832467

[45] Patel SR Ayas NT Malhotra MR White DP Schernhammer ES Speizer FE. A prospective study of sleep duration and mortality risk in women. Sleep (2004) 27:440–4. doi: 10.1093/sleep/27.3.440 15164896

[46] Zhang Y Punnett L Gore R The CPHNEW Research Team. Relationships among employees’ working conditions, mental health, and intention to leave in nursing homes. J Appl Gerontol (2014) 33:6–23. doi: 10.1177/0733464812443085 24652941 PMC11837970

[47] Waldhauser F Vierhapper H Pirich K. Abnormal circadian melatonin secretion in night shift workers. New Engl J Med (1986) 315:1614–5. doi: 10.1056/NEJM198612183152516 3785329

[48] Teixeira KRC Dos Santos CP de Medeiros LA Mendes JA Cunha TM De Angelis K. Night workers have lower levels of antioxidant defenses and higher levels of oxidative stress damage when compared to day workers. Sci Rep (2019) 9:4455. doi: 10.1038/s41598-019-40989-6 30872663 PMC6418308

[49] Meier-Ewert HK Ridker PM Rifai N Price N Dinges DF Mullington JM. Absence of diurnal variation of c-reactive protein concentrations in healthy human subjects. Clin Chem (2001) 47:426–30. doi: 10.1093/clinchem/47.3.426 11238292

[50] Haack M Kraus T Schuld A Dalal M Koethe D Pollmächer T. Diurnal variations of interleukin-6 plasma levels are confounded by blood drawing procedures. Psychoneuroendocrinology (2002) 27:921–31. doi: 10.1016/S0306-4530(02)00006-9 12383453

[51] Zheng H Patel M Hryniewicz K Katz SD. Association of extended work shifts, vascular function, and inflammatory markers in internal medicine residents: a randomized crossover trial. J Am Med Assoc (2006) 296:1049–50. doi: 10.1001/jama.296.9.1049 16954481

[52] Johnson CY Tanz LJ Lawson CC Schernhammer ES Vetter C Rich-Edwards JW. Night shift work and cardiovascular disease biomarkers in female nurses. Am J Ind Med (2020) 63:240–8. doi: 10.1002/ajim.23079 PMC8572536 31828843

[53] Knutsson A. Health disorders of shift workers. Occup Med (Lond) (2003) 53:103–8. doi: 10.1093/occmed/kqg048 12637594

[54] Scheer FA Hilton MF Mantzoros CS Shea SA. Adverse metabolic and cardiovascular consequences of circadian misalignment. Proc Natl Acad Sci US A (2009) 106:4453–8. doi: 10.1073/pnas.0808180106 PMC2657421 19255424

[55] Kervezee L Cermakian N Boivin DB. Individual metabolomic signatures of circadian misalignment during simulated night shifts in humans. PloS Biol (2019) 17(6):e3000303. doi: 10.1371/journal.pbio.3000303 31211770 PMC6581237

[56] Faraut B Boudjeltia KZ Vanhamme L Kerkhofs M. Immune, inflammatory and cardiovascular consequences of sleep restriction and recovery. Sleep Med Rev (2012) 16:137–49. doi: 10.1016/j.smrv.2011.05.001 21835655

[57] Cappuccio FP D’Elia L Strazzullo P Miller MA. Quantity and quality of sleep and incidence of type 2 diabetes: A systematic review and meta-analysis. Diabetes Care (2010) 33:414–20. doi: 10.2337/dc09-1124 PMC2809295 19910503

[58] Cappuccio FP Cooper D D’Elia L Strazzullo P Miller MA. Sleep duration predicts cardiovascular outcomes: A systematic review and meta-analysis of prospective studies. Eur Heart J (2011) 32:1484e92. doi: 10.1093/eurheartj/ehr007 21300732

[59] Ogilvie RP Patel SR. The epidemiology of sleep and obesity. Sleep Health (2017) 5:383–8. doi: 10.1016/j.sleh.2017.07.013 PMC5714285 28923198

[60] Xing Z Gauldie J Cox G Baumann H Jordana M Lei XF. IL-6 is an antiinflammatory cytokine required for controlling local or systemic acute inflammatory responses. J Clin Invest (1998) 101:311–20. doi: 10.1172/JCI1368 PMC508569 9435302

[61] Tilg H Dinarello CA Mier JW. IL-6 and APPs: anti-inflammatory and immunosuppressive mediators. Immunol Today (1997) 18:428–32. doi: 10.1016/S0167-5699(97)01103-1 9293158

[62] Padgett D Glaser R. How stress influences the immune response. Trends Immunol (2003) 24:444–8. doi: 10.1016/S1471-4906(03)00173-X 12909458

[63] Castanon-Cervantes O Wu M Ehlen JC Paul K Gamble KL Johnson RL. Dysregulation of inflammatory responses by chronic circadian disruption. J Immunol (2010) 185:5796–805. doi: 10.4049/jimmunol.1001026 PMC2974025 20944004

[64] Loef B Nanlohy NM Jacobi RHJ van de Ven C Mariman R van der Beek AJ. Immunological effects of shift work in healthcare workers. Sci Rep (2019) 9(1):18220. doi: 10.1038/s41598-019-54816-5 31796836 PMC6890754

[65] Lange T Perras B Fehm HL Born J. Sleep enhances the human antibody response to hepatitis a vaccination. Psychosom Med (2003) 65:831–5. doi: 10.1097/01.PSY.0000091382.61178.F1 14508028

[66] Prather AA Janicki-Deverts D Hall MH Cohen S. Behaviorally assessed sleep and susceptibility to the common cold. Sleep (2015) 38:1353–9. doi: 10.5665/sleep.4968 PMC4531403 26118561

[67] Patel SR Malhotra A Gao X Hu FB Neuman MI Fawzi WW. A prospective study of sleep duration and pneumonia risk in women. Sleep (2012) 35:97–101. doi: 10.5665/sleep.1594 22215923 PMC3242694

[68] Ruiz FS Rosa DS Zimberg IZ Dos Santos Quaresma MV Nunes JO Apostolico JS. Night shift work and immune response to the meningococcal conjugate vaccine in healthy workers: a proof of concept study. Sleep Med (2020) 75:263–75. doi: 10.1016/j.sleep.2020.05.032 32866895

[69] Eckel RH Depner CM Perreault L Markwald RR Smith MR McHill AW. Morning circadian misalignment during short sleep duration impacts insulin sensitivity. Curr Biol (2015) 25:3004–10. doi: 10.1016/j.cub.2015.10.011 26549253

[70] Bandín C Scheer FA Luque AJ Ávila-Gandía V Zamora S Madrid JA. Meal timing affects glucose tolerance, substrate oxidation and circadian-related variables: A randomized, crossover trial. Int J Obes (2015) 39:828–33. doi: 10.1038/ijo.2014.182 25311083

[71] Marinac CR Sears DD Natarajan L Gallo LC Breen CI Patterson RE. Frequency and circadian timing of eating may influence biomarkers of inflammation and insulin resistance associated with breast cancer risk. PloS One (2015) 10(8):e0136240. doi: 10.1371/journal.pone.0136240 26305095 PMC4549297

[72] Wirth MD Zhao L Turner-McGrievy GM Ortaglia A. Associations between fasting duration, timing of first and last meal, and cardiometabolic endpoints in the national health and nutrition examination survey. Nutrients (2021) 13(8):2686. doi: 10.3390/nu13082686 34444846 PMC8397975

[73] Zhang Y Cordina-Duverger E Komarzynski S Attari AM Huang Q Aristizabal G. Digital circadian and sleep health in individual hospital shift workers: A cross sectional telemonitoring study. e-BioMedicine; (2022) 81:104121. doi: 10.1016/j.ebiom.2022.104121 PMC9253495 35772217

