# Supplementary figures and images for: Immune disruptions and night shift work in hospital healthcare professionals: The intricate effects of social jet-lag and sleep debt
Source: Front Immunol. 2022 Sep 9;13:939829. doi: 10.3389/fimmu.2022.939829 (PMC9509137; doi:10.3389/fimmu.2022.939829)

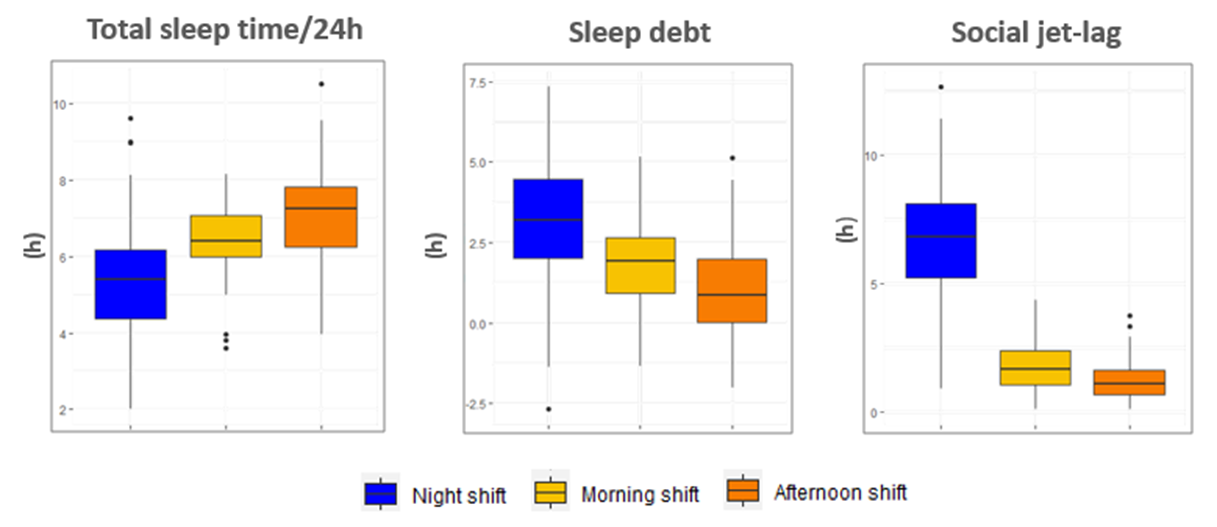

Supplement: Supplementary Figure 1 — Total sleep time, sleep debt and social jet-lag according to the type of shift. Total Sleep Time (TST) per 24 h on work days (based on the typical hours reported on sleep diary on work days), sleep debt (TST per 24 h on free days – TST per 24 h on working days) and social jet-lag status (difference between the average mid-sleep point from the main sleep episode on free days and that on workdays) are shown here as medians (25%–75% range). [file Image_1.tif]
